# Initial stages of oxide formation on the Zr surface at low oxygen pressure: An in situ FIM and XPS study

**DOI:** 10.1016/j.ultramic.2015.02.016

**Published:** 2015-12

**Authors:** I. Bespalov, M. Datler, S. Buhr, W. Drachsel, G. Rupprechter, Y. Suchorski

**Affiliations:** Institute of Materials Chemistry, Vienna University of Technology, Getreidemarkt 9, 1060 Vienna, Austria

**Keywords:** Zirconium, Zirconia, Surface oxidation, X-Ray photoelectron spectroscopy, Field ion microscopy

## Abstract

An improved methodology of the Zr specimen preparation was developed which allows fabrication of stable Zr nanotips suitable for FIM and AP applications. Initial oxidation of the Zr surface was studied on a Zr nanotip by FIM and on a polycrystalline Zr foil by XPS, both at low oxygen pressure (10^−8^–10^−7^ mbar). The XPS data reveal that in a first, fast stage of oxidation, a Zr suboxide interlayer is formed which contains three suboxide components (Zr^+1^, Zr^+2^ and Zr^+3^) and is located between the Zr surface and a stoichiometric ZrO_2_ overlayer that grows in a second, slow oxidation stage. The sole suboxide layer has been observed for the first time at very early states of the oxidation (oxygen exposure ≤4 L). The Ne^+^ FIM observations are in accord with a two stage process of Zr oxide formation.

## Highlights

• A new methodology of the Zr specimen preparation for FIM and AP applications.
• Zr suboxide interlayer formation between the Zr surface and a stoichiometric ZrO_2_.
• The sole suboxide layer has been observed at very early stages of the Zr oxidation.

## 1 Introduction

When a “freshly-prepared” metal or semiconductor surface is exposed to an oxygen-containing atmosphere, an oxide layer is formed even at low temperatures and at low oxygen pressures. This phenomenon is of fundamental technological importance in different fields, such as catalysis, medical, biomechanical and electronic devices, fuel cell technology and photonics [1], [2], [3], [4], [5]. Increasing miniaturization of devices and sensors boosts the importance of the oxide-film contribution since the relative impact of the surface layer increases when downsizing metal/oxide structures [6], [7].

However, the number of systems, for which a (highly desirable) atomic scale control over the oxidation processes is achieved, is still scarce and the amount of knowledge on the microscopic mechanisms of the reactions between adsorbed oxygen and solid surfaces is thus rather limited [8], [9]. This is due to the chameleon-like changing character of the oxygen-surface interactions occurring on the nanoscale, which hampers a direct experimental observation. In addition, the frequent occurrence of metastable oxide structures makes difficult the use of equilibrium phase diagrams and other thermodynamic data for explanation or prediction of the atomistic mechanisms of surface oxidation [10], [11], [12].

To describe the surface oxidation of metals, a number of models has been developed, such as the Cabrera–Mott's [13] and Fromhold’s [14] models, which were continuously modified during the years [15], [16], [17]. However, these models assume a uniform oxide growth governed by the transport of species through the continuous oxide film and thus do not account for the inhomogeneities in the chemical composition, structure and morphology across the oxide layer. This stimulates extensive experimental studies both for the practical use and for the development of refined models.

Thin native oxide films formed on zirconium and zirconium–alloy surfaces are of particular interest, since they are usually dense enough to passivate the surface against the further incorporation of reactive gases into the bulk. This allows using such surfaces in high-temperature corrosive atmospheres, e.g. in nuclear reactors, but also still requires detailed knowledge on the kinetics and mechanism of growth of zirconium oxide films for understanding of passivation mechanisms [18].

Despite of the general agreement on the processes which precede oxide formation, i.e. subsurface oxygen formation, dissolution of oxygen atoms in the zirconium lattice and ordering of the O-superlattice in the subsurface layer [19], [20], there is less consent on the role of suboxides in the initial Zr oxidation. Although earlier XPS studies [21], [22], [23] have already suggested that suboxides may be formed upon low oxygen exposures (0.5–2 L), the mutual proportion and even the number of the appearing suboxides are still debated. There is only agreement that the native oxide growth occurs as a two stages process where the first rapid stage of oxidation (characterized by Zr enrichment and oxygen deficiency) occurs in the region between the metal surface and the ZrO_2_ overlayer, followed by a slower stage of ZrO_2_ formation [24], [25]. Moreover, in most studies the presence of suboxides is still neglected presumably due to their minor contribution to the total oxide amount. The reason is mainly the “masking” role of a much thicker ZrO_2_ overlayer grown on the ultrathin suboxide interlayer, the sole suboxide layer was to our knowledge not yet observed, supposedly due to the very high rate of initial oxidation. Using the 1D atom-probe (1D-AP) technique, the “masking” problem can be circumvented and in fact, the indications were found for a composition similar to ZrO located between the ZrO_2_ overlayer and the metal surface [26]. Applying the most advanced 3D-AP with laser pulsing, this finding was confirmed, however, possible miniature fractures between the oxide and the metal of the needle-shape specimen make the analysis of the transition region difficult [27]. In addition, possible grain boundaries increase the fragility of the atom-probe samples and adulterate the AP-data. Such high fragility of Zr specimens under high electric field stress hampered for a long time AP and FIM studies of Zr and Zr oxides. Thus it is highly challenging to develop a methodology for stable Zr tips fabrication for AP and FIM studies as well as to obtain reliable data on the initial oxidation of Zr.

In the present contribution we present a new method for Zr tips fabrication based on the fragmented recrystallization of a Zr wire as a result of repeated “hcp-to-bcc” and vice versa phase transitions, and the first FIM observation of Zr oxidation on a microscopic-scale. For comparison, an XPS study of the initial oxidation on polycrystalline Zr foil with emphasis on the identification of Zr suboxides was performed.

## 2 Experimental

The experiments were performed in two independent bakeable all-metal UHV setups with a base pressure of ≤10^−9^ mbar: (i) an FIM system which contains a tip assembly, allowing operation in a controlled temperature range of 78–900 K*,* a channel plate, a gas-supply system where high purity gases (Ne for FIM imaging and oxygen for Zr oxidation) can be supplied via leak-valves under mass-spectrometric control, and (ii) an XPS system with a Phoibos 100 hemispherical energy analyzer and XR 50 twin anode X-ray source (both *SPECS*, Germany). The XPS system is also equipped with gas supply facility and, additionally, the sample can be cleaned by argon ion sputtering. More details of the experimental setups can be found elsewhere ([28] for XPS and [29] for FIM/FEM).

**Fig. 1:**
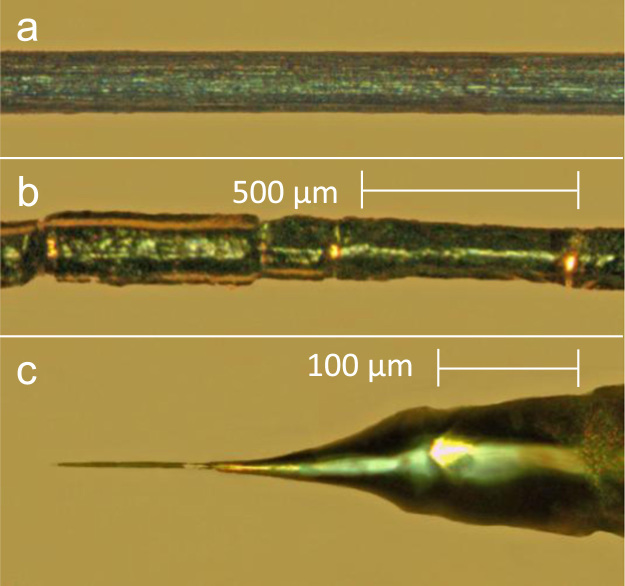
Fabrication of a stable Zr nanotip. a) primary material: commercial Zr wire, 0.127 mm diameter, *Alfa Aesar*; b) the same wire after annealing at 973 K for several hours and after the formation of small monocrystals at 1173 K; c) a Zr nanotip fabricated by electro polishing from the monocrystal visible at right side of the Fig. 1b.

As already mentioned, an improved methodology for the Zr tip preparation was elaborated in present work, based on the supposition that the low stability limit of Zr nanotips in previous studies originates from the small size of the crystallites. To increase the size of crystallites, a Zr wire (0.127 mm diameter, *Alfa Aesar,* Fig. 1a) was preliminarily annealed in UHV under mechanical tension. After conventional annealing at 973 K for several hours, as used in most other studies for Zr sample preparation, the surface became clearly smoother. During the further annealing at 1173 K for 4 h, fragments of small monocrystals (~0.3 mm, Fig. 1b) were formed as a result of the “hcp-to-bcc” phase transition at 1143 K accompanying the recrystallization. This was clearly visible as a drop in the (continuously monitored) electrical resistance despite of the increasing temperature. The reverse “bcc–to–hcp” transition was performed via slowly cooling down the annealed Zr wire, in order to prevent the distortions and corrugations resulting from the martensitic-like transition. At cooling rates lower than 25 K/min we succeeded in conserving the fragmented structure and smooth surface down to room temperature.

The Zr nanotips were then prepared from the mono-crystalline fragments of such an annealed and recrystallized Zr wire via two stage electro polishing. In the first stage we used a DC “dip etching” at 15 V/150 mA with a H_2_O/HClO_4_/CH_3_COOH mixture of the ratio 10/15/75. In the final stage the DC microscope-zone-polishing at 0.1–5 V DC by H_2_O/HClO_4_/2-butoxy-ethanol of the ratio 1/1/98 was used.

To compare the nm- and μm- scaled samples, a polycrystalline Zr foil was used for the XPS measurements (10×10 mm^2^, thickness 0.2 mm, MaTeck, 99.8% purity). The foil sample was thoroughly polished before to be mounted on a standard *SPECS/Omicron* sample holder equipped with the thermocouple contacts.

The temperature of the specimen was measured by a NiCr/Ni thermocouple spot-welded to the front side of the foil. To minimize the contamination by segregating Fe (usual contamination in Zr foils) several standard UHV cleaning procedures, namely Ar^+^ ion sputtering (Ar pressure 10^−^^5^ mbar, *E*_kin_=1.7 keV), following by annealing at 1173 K was performed under XPS control. At the last stage, the foil sample was subjected to exactly the same thermal treatment as Zr wire to ensure the same surface condition as on the Zr tip.

## 3 Results and discussion

### 3.1 FIM observations

**Fig. 2:**
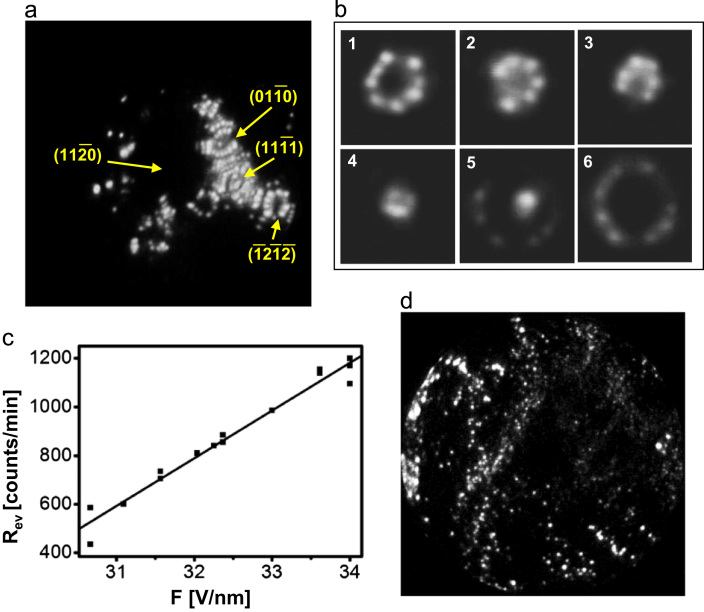
FIM observations of Zr tip oxidation. a) Ne^+^ field ion image at 77 K and 33 V/nm under conditions of intensive field evaporation. The dark region in the $\left(11\bar{2}0\right)$ region is due to the probe-hole in the channelplate; b) a sequence of video-frames illustrating field evaporation of the $\left(\overset{¯}{1}2\overset{¯}{1}\overset{¯}{2}\right)$ facet; c) field dependence of the evaporation rate at 77 K; d) the same as in (a) but after oxidation at 300 K and 10^−8^ mbar.

Because of the high fragility of Zr specimens, FIM imaging of the Zr surface with Ne^+^ ions (at ~35 V/nm) is a difficult task, in addition, the evaporation field of Zr at e.g. 77 K ranges below of the BIF (best image field) value for Ne. The only published FIM image of Zr with atomic resolution is that by Carroll and Melmed at 30 K [30], i.e. far below the liquid nitrogen temperature. Nevertheless, using the new preparation technique, a sufficient stability of Zr specimens was achieved for the Ne^+^ FIM imaging at 77 K. Fig. 2a shows a Ne^+^ FIM image obtained at applied field of 33 V/nm during continuous field evaporation. A set of the video-frames (Fig. 2b), monitoring the field evaporation of the $\left(\overset{¯}{1}2\overset{¯}{1}\overset{¯}{2}\right)$ facet and Fig. 2c showing the dependence of the Zr evaporation rate at 77 K on the applied electric field, illustrate the stability of the Zr sample in a wide range of applied electric field. Although at the instant of field evaporation, as was shown by earlier DFT calculations [31], the positions of the surface (imaged) atoms can slightly change along the applied field direction (i.e. perpendicular to the surface), the acquired FIM images are still 2D projections with unchanged lateral distances, i.e. they reflect the correct geometry of the surface.

The interaction of the clean Zr surface with oxygen at 10^−8^ mbar (as introduced in field free conditions at 300 K) results even at very low exposures in a drastic rearrangement of the surface layer, visible as formation of ridges and enlarging of the facet sizes (Fig. 2b). At exposures less than 4 L, the original “metallic” surface could be repeatedly restored by field evaporation. In turn, higher exposition leads to the formation of a rather “structureless” oxide layer with high work function (presumably ZrO_2_) which could not be removed by electric field up to 42 V/nm at 77 K. Further increase of the applied field causes a fracture of the specimen, likely at the metal–oxide interface.

Apparently, at the earlier stage of oxidation, a suboxide structure is formed with an enrichment by Zr atoms which facilitate the process of field evaporation due to the local field enhancement over the positively charged metal atoms [31]. In turn, the enrichment by negatively charged oxygen atoms in a ZrO_2_ layer weakens the driving force acting at individual atoms and thus hinders the field evaporation. This leads to the necessity to apply much higher fields which may excess the field stress limit. Therefore, the use of the AP with laser pulsing seems to be the method of choice for ZrO_2_ studies.

It has to be noted, that the remarkable microscopic structure changes occurring during the first stage of Zr oxidation (compare Fig. 2a and 2b) are significant on the Angström-scale, whereas for the planar macroscopic surfaces used for XPS studies, they are rather negligible in the sense of surface relief modifications.

### 3.2 XPS results

**Fig. 3:**
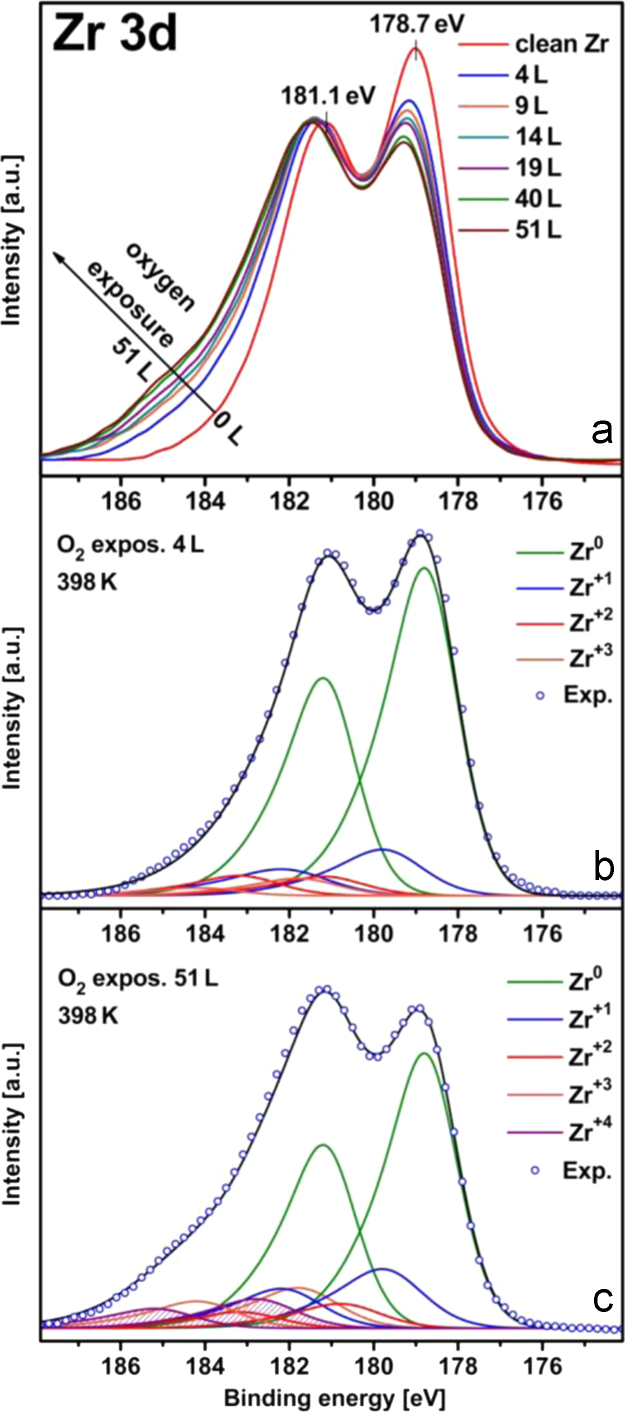
In situ XPS of the initial stages of Zr oxidation. (a) set of Zr 3d spectra at different oxygen exposures in the range 4–51 L at p_O2_=2×10^−8^ mbar, *T*=398 K; (b) deconvolution of the spectrum taken at 4 L. Apart from metallic Zr only the suboxide contribution is visible (Zr^+1^, Zr^+2^ and Zr^+3^ are present); (c) the same, but for exposure of 51 L, the ZrO_2_ contribution is clearly visible (shaded area), apart from suboxides.

To characterize Zr suboxides formed during the first rapid state of initial Zr oxidation, *in situ* XPS measurements were performed at constant oxygen pressure of 2×10^−8^ mbar for oxygen exposures in the range 4–51 L. Fig. 3 shows the corresponding XPS spectra for the Zr 3d region: the spectrum taken for 4 L exposure (Fig. 3b) exhibits solely the suboxide contribution, apart from metallic Zr, to our knowledge this is the first XPS observation of this kind. Interestingly, already at such low exposure three suboxide components Zr^+1^, Zr^+2^ and Zr^+3^ are present. Spectra taken at higher oxygen exposure, see e.g. Fig. 3c, show, in addition to suboxides, also ZrO_2_ contribution, a usual observation during the initial oxidation of Zr. We are aware of the fact that such *in situ* measurements reflect a thermodynamic situation which is far from the equilibrium and the sole suboxide layer formed at the very beginning of the oxidation process might undergo an evolution, transforming into the ZrO_2_ overlayer and a suboxide layer depleted of oxygen below. At the moment it is however hardly possibly to find evidences for such transformation, because even after the oxygen valve is closed the oxidation process proceeds at very low partial “residual oxygen” pressures <10^−9^ mbar.

**Fig. 4:**
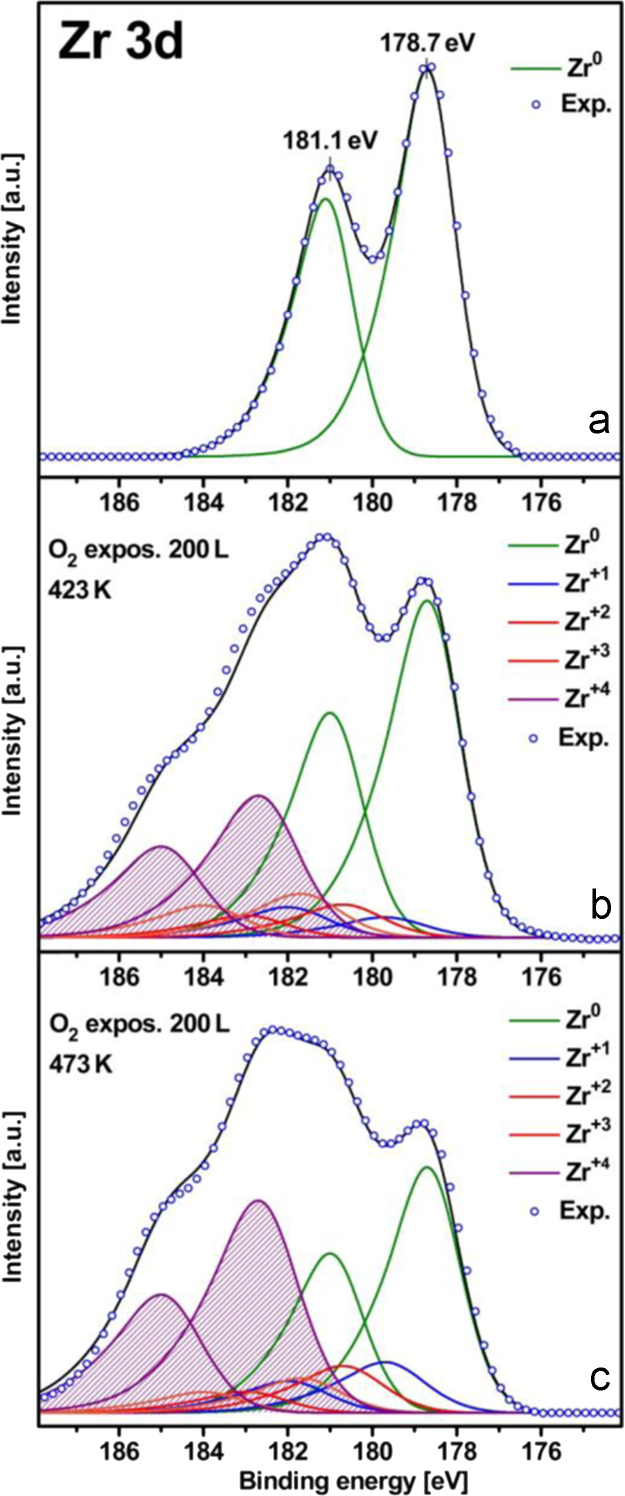
Zirconium oxidation at different temperatures. (a) Zr 3d XPS spectra of the clean surface before the oxidizing oxygen treatment; (b) the same but after oxidation at constant oxygen pressure of 8×10^−7^ mbar at 423 K (200 L); (c) the same as in (b) but at 473 K. Shaded area: ZrO_2_ contribution.

To follow the second, slower stage of Zr oxidation, XPS measurements were performed at constant oxygen pressure of 8×10^−7^ mbar for different temperatures, with the measurements always starting with the clean (sputtered, annealed and XPS controlled) Zr surface. Fig. 4a–c shows the corresponding XPS spectra for the Zr 3d region measured for the clean Zr foil and for the same foil oxidized at 423 K and 473 K, respectively. The spectra again clearly show the presence of three suboxides with the Zr^1+^, Zr^2+^, Zr^3+^ components, apart from ZrO_2_.

The presence of three suboxide components is in agreement with the XPS and AES results of Morant et al. [21] and Nishino et al. [32] who also detected three suboxides (two suboxides were found in earlier studies [23], [33]. The absolute values for the 3d5/2 Zr^0^ peak (178.7 eV) and the spin–orbit splitting between the 3d5/2 and 3d3/2 peaks (2.4 eV) also correspond well with those studies.

Although the presence of the suboxide components was already reported earlier [21], [22], [23], [24], [25], [32], [33], questions about the existence of a compact suboxide interlayer (the suboxides might also create nanosized inclusions within the ZrO_2_ overlayer or even be more or less homogeneously distributed) and about its thickness are hardly discussed in the literature, apart from few recent publications [24], [25]

It is, however, possible to determine the thickness of the oxide (suboxide) layer from the Zr 3d spectra. Usually, for a moderate oxide layer thicknesses (<5 nm), Zr 3d signals originating both from the oxide layer and from the Zr substrate can be detected. Although the cumulative XPS signal contains in principle all information about the oxide layer thickness, straightforward distillation of such data is, unfortunately, complicated. In the case of a flat homogeneous substrate covered by a uniform oxide overlayer with an intermediate layer of differing thickness, the relation of the signals originating from the substrate and both layers can be calculated using the fact that the “substrate signal” originating from the metal is exponentially attenuated by both oxide layers prior to detection. In turn, the signal from the suboxide interlayer is attenuated by the topmost ZrO_2_ overlayer [34].

**Fig. 5:**
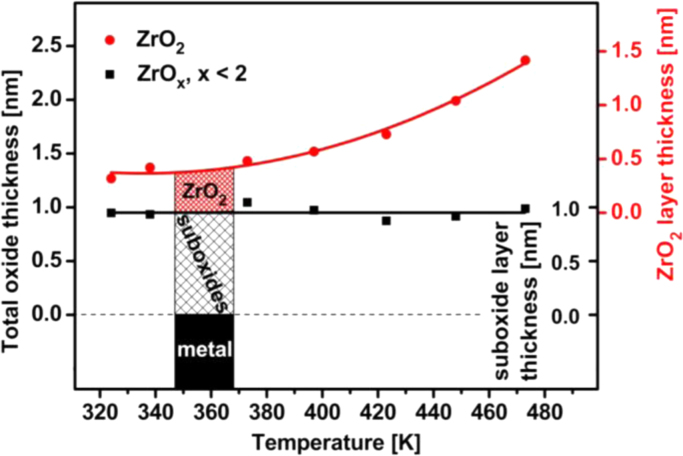
Thickness of the ZrO*_x_* interlayer, ZrO_2_ overlayer and total Zr oxide layer thickness as obtained from XPS measurements at different temperatures. Results for the 423 K and 473 K are taken from the data shown in Fig. 3. The inset shows schematically the two-layer model.

The set of corresponding equations (see e.g. Appendix in Ref. [35]) provides the thickness of the ZrO_2_ layer and of the ZrO_*x* (*x*<2)_ layer, shown in Fig. 5 as a dependence on the oxidizing temperature (for constant oxidation time and oxygen pressure).

As clearly visible from Fig. 5, the thickness of the suboxide interlayer remains nearly constant, whereas the overlying ZrO_2_ film grows continuously. Although such an evaluation does not account for elastic scattering effects, in the present case such a contribution would hardly change the final result.

The present XPS results are in agreement with the existing consensus that the initial oxidation of Zr comprises two stages: (i) a very fast migration of Zr cations into the oxide layer, which is caused mainly by the Mott potential formed between the Fermi level of the metal substrate and the acceptor levels of oxygen species on the oxide surface, and (ii) a much slower oxide-film growth, induced by coupled electron- and cations/anions-current due to the kinetic potentials [17], [35], [36]. At this stage the oxygen ion diffusion through the oxide layer might contribute significantly [37], [38]. The oxygen atoms, as noticed already in the early work by Wang et al. [39], may also penetrate into the bulk of Zr even at room temperature, occupying tetrahedral states in the *hcp* lattice of Zr. All these processes contribute to the formation of Zr- and oxygen-concentration gradients, thus forming a suboxide layer between the metal surface and the ZrO_2_ overlayer.

## 4 Conclusions and outlook

An improved methodology of the Zr specimen preparation allows fabrication of stable Zr nanotips suitable for the FIM and AP studies. Despite of the relatively low values of the evaporation field for Zr it appears to be possible to image the Zr surface with Ne^+^ ions at 77 K, of course under conditions of continuous field evaporation. The initial stages of Zr oxidation at low oxygen pressures (10^−8^–10^−7^ mbar) were observed by FIM for a Zr nanotip and studied in detail by XPS for a polycrystalline Zr foil. The XPS data confirm the two stages model of initial Zr oxidation and reveal the formation of three-component suboxides as an interlayer between the metal surface and a stoichometric ZrO_2_ overlayer. Both, the FIM observations and XPS studies are in agreement with a two stage process of Zr oxide formation.

The sole suboxide layer was observed by XPS for the first time as a transient state at oxygen exposures ≤4 L. The measurements indicate a concentration gradient of Zr and/or oxygen through the suboxide interlayer, with stoichiometric ZrO_2_ at its outer side and increasing enrichment in Zr towards the metal substrate surface.

To reveal the atomic details of the Zr suboxide formation, further FIM studies, particularly in combination with modern AP-techniques with laser pulsing are necessary and seem to be promising in view of the possibility to fabricate stable Zr nanotip-specimens.
